# Total Testosterone as a Specific Marker of Acute Kidney Injury in Male Patients With Myocardial Infarction

**DOI:** 10.7759/cureus.28682

**Published:** 2022-09-01

**Authors:** Niya E Semerdzhieva, Adelina D Tsakova, Mariana Gospodinova, Simeon I Dimitrov, Stefan Denchev

**Affiliations:** 1 Emergency Department, National Heart Hospital, Sofia, BGR; 2 Central Clinical Laboratory, Medical University of Sofia, Aleksandrovska University Hospital, Sofia, BGR; 3 Cardiology Department, University Hospital St. Ivan Rilski, Sofia, BGR; 4 Invasive Cardiology Department, Hristo Botev Hospital, Vratsa, BGR; 5 Cardiology Department, Medical Center 'Mediva', Sofia, BGR

**Keywords:** c-reactive protein, inflammation, total testosterone, myocardial infarction, acute kidney injury

## Abstract

Aim

The aim of the present study was to assess the significance of total testosterone (T) as a marker of acute kidney injury (AKI) in patients with acute myocardial infarction (MI).

Patients and methods

The study was a retrospective, single-center cohort study that included 55 consecutive male patients diagnosed with acute MI who were admitted to the Cardiology Clinic of Alexandrovska University Hospital (Sofia, Bulgaria) between July 2011 and December 2013. The plasma total T levels, measured at admission, the peak levels of myocardial necrosis markers, high-sensitive C-reactive protein (hsCRP), and the left ventricular ejection fraction (LVEF) were analyzed in relation to the incidence of AKI.

Results

The occurrence of AKI was positively predicted by reduced EF (OR=0.825; CI=0.724-0.942; P=0.004), advanced age (OR=1.077; CI=1.038-1.151; P=0.029), and low levels of total T (OR=0.837; CI=0.707-0.990; P=0.037). Reduced systolic function (OR=0.861; 95% CI=0.758-0.978; P=0.022 for EF) and marginally age (OR=1.094; 95% CI=1.000-1.197; P=0.051) contributed to the incidence of AKI in a multivariate model. Total T was not an independent factor (OR=0.841; 95% CI=0.669-1.058; P=0.139) for AKI. The total T levels were significantly inversely correlated with the peak of hsCRP (r= -0.153; P=0.009) and showed a tendency to inverse relation with the SYNTAX score (r= -0.235; P=0.083).

Conclusion

The total T levels are significantly inversely related to the peak of hsCRP and as a tendency to the SYNTAX score in male patients with acute MI. A low level of plasma total T is not an independent marker of AKI in acute MI. Advanced age and low EF are independent factors for AKI discrimination in a small cohort of patients with acute MI.

## Introduction

Acute kidney injury (AKI) is often exhibited by patients with an acute illness. The incidence of AKI following the usage of contrast media in therapeutic procedures, as is the percutaneous coronary intervention (PCI) in acute myocardial infarction (MI), is 26% [1]. Of all AKI cases, 30% are considered preventable [2]. Examples of preventive measures are intravenous hydration in high-risk patients using 0.9% sodium chloride or isotonic sodium bicarbonate. Predictive models with a generally good negative predictive value оf risk have been validated to identify patients at risk for AKI in the acute phase of MI who are undergoing percutaneous intervention [1, 3]. A consensus statement from the Acute Disease Quality Initiative recommended using validated biomarkers and encouraged research on novel, specifically non-kidney, biomarkers to identify patient populations at risk for AKI for whom preventive interventions have been shown to improve outcomes [1]. Previous studies have reported conflicting results about the significance of sex, estradiol, and androgens in modifying the severity of renal ischemia-reperfusion injury (IRI) [4, 5]. Males are apparently much more susceptible to ischemia/reperfusion-induced kidney injury [4, 5]. The aim of the present study was to assess the significance of total testosterone (T) as a marker of AKI in patients with acute MI treated preliminarily with percutaneous coronary intervention.

## Materials and methods

Patients

The present study was a retrospective, single-center cohort study that included 55 consecutive male patients diagnosed with acute MI who were admitted to the Cardiology Clinic, Alexandrovska University Hospital (Sofia, Bulgaria) between July 2011 and December 2013. It is a subanalysis of an investigation of the relationship of sex steroids with inflammation in the acute phase of coronary syndrome. Written informed consent according to the Declaration of Helsinki was obtained from each patient before enrollment. The study did not include experimental or novel treatment. The Ethics Committee of the Medical University of Sofia (Sofia, Bulgaria) confirmed that the study design and methods were relevant to the ethical standards necessary when conducting research involving humans (approval No 68/05.05.2012). Exclusion criteria for the study included acute and chronic inflammatory processes, current use of antibiotics, surgery or traumatic event within two weeks of admission and during the hospital stay, known or suspected neoplastic disease, and intake of immunomodulating or hormone-level modulating (including hormone replacement) therapy.

Methods

AKI was defined according to the Kidney Disease: Improving Global Outcomes (KDIGO) criteria as a 25% decrease in the estimated glomerular filtration rate (GFR) within seven days of kidney stress [6]. The GFR was estimated from the serum creatinine levels using the equation based on the results of the Modification of Diet in Renal Disease study [7].

Measurements of plasma total T at admission, and peak levels of myocardial necrosis markers (creatine kinase [CK]), the CK isoenzyme MB (CK-MB), and high-sensitive cardiac troponin T (hsTnT)] and high-sensitive C-reactive protein (hsCRP) were carried out. The left ventricular ejection fraction (LVEF) was used as a marker of left ventricular systolic function. It was evaluated in all patients during the acute phase via the biplane Simpson’s method using 2D echocardiography [8]. The SYNTAX score (incorporates the number and the location of the obstructive atherosclerotic coronary lesions) was calculated in each patient at coronary angiography and served as an objective measure of the severity of coronary atherosclerosis.

The T and hsTnT levels in plasma were assessed by electrochemiluminescent immunoassay with commercially available reagent labeled as Elecsys Testosterone II and Elecsys Troponin T-high sensitive (TnT-hs) from Roche Diagnostics GmbH in an automated Elecsys 2010 analyzer (Roche Diagnostics GmbH). Briefly, the test consisted of a two-step incubation of the sample with a biotinylated monoclonal hormone-specific antibody (Elecsys Testosterone II, Roche Diagnostics GmbH, Mannheim, Germany), a mixture of streptavidin-coated microparticles and a T derivate. The troponin assay was based on the sandwich principle; the sample is incubated with a biotinylated monoclonal cardiac troponin T‑specific antibody, and a monoclonal cardiac troponin T‑specific antibody labeled with a ruthenium complex reacts to form a complex. After the addition of streptavidin-coated microparticles, the complex formed becomes bound to the solid phase via the interaction of biotin and streptavidin. In both of the tests, the microparticles formed were magnetically captured onto the surface of an electrode in a measuring cell. Application of a voltage to the electrode induced chemiluminescent emission, which was measured by a photomultiplier.

CRP concentrations were measured with a latex-enhanced immunoturbidimetric assay in a COBAS INTEGRA 400 analyzer (Roche Diagnostics GmbH, Mannheim, Germany). The test is based on the agglutination of latex particles coated with anti-human CRP antibodies (Roche Diagnostics GmbH, Mannheim, Germany). The precipitate was measured by turbidimetry [9].

Statistical analysis

The results are presented as mean ± SD, and were analyzed using parametric (unpaired Student’s t-test and Pearson correlation coefficient) and nonparametric (χ2, Fisher’s exact test, Mann-Whitney U test, and Spearman's rank-order correlation) methods using SPSS version 22 (IBM Corp., Armonk, NY). P<0.05 was considered to indicate a statistically significant difference.

## Results

The baseline characteristics are presented in Table 1.

**Table 1:** Characteristics of the study group. CK: Creatinine phosphokinase; CK-MB: MB isoenzyme of creatinine phosphokinase; hsTnT; High-sensitive cardiac troponin T; hsCRP: High-sensitive C-reactive protein; GFRbaseline: Glomerular filtration rate at presentation; GFRmin: Peak reduction in glomerular filtration rate.

Variables	Patients, n = 55
Acute kidney injury, n (%)	13 (17.1)
Age, years	62.5±12.2
Diabetes mellitus, n (%)	32 (42.1)
Anemia, n (%)	17 (23.6)
Statin, n (%)	12 (22.6)
Β-blocker, n (%)	15 (27.3)
Testosterone, nmol/l	13.6±5.4
CK, U/l	1,239.8±1,587.4
CK-MB, U/I	135.8±187.8
hsTnT, ng/l	2.3±3.0
hsCRP, mg/l	36.1±45.9
WBC, x10^9^/l	10.3±3.5
Ejection fraction, %	52.2±8.7
SYNTAX score	16.1±10.0
GFR_baseline_,ml/min/1.73 m^2^	76.0±20.1
GFR_min_, ml/min/1.73 m^2^	71.4±21.8
Angiographic dye, ml	285.9±130.7

**Figure 1:**
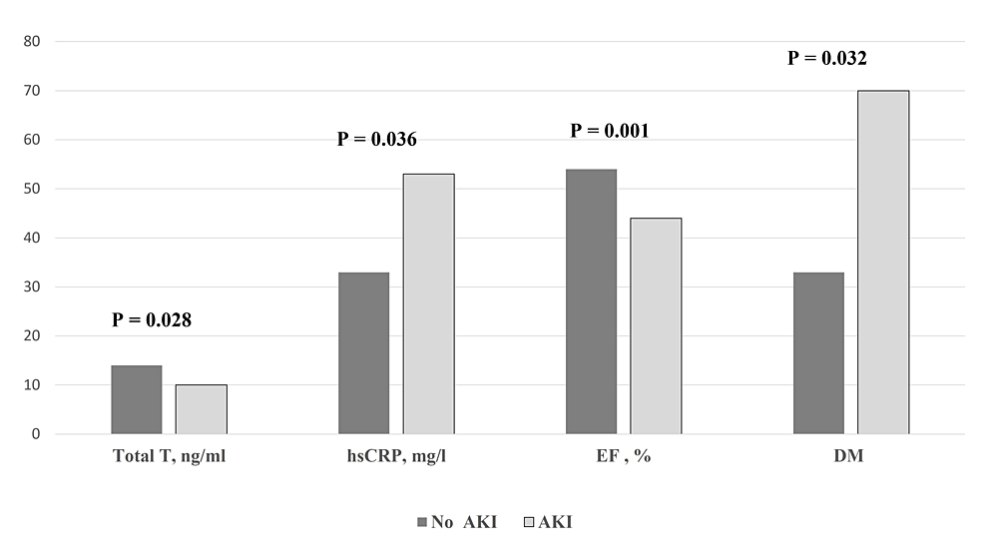
Variables associated with the occurrence of AKI in myocardial infarction. T: Testosterone; hsCRP: High-sensitive C-reactive protein; EF: Ejection fraction; DM: Diabetes mellitus; AKI: Acute kidney injury.

**Figure 2:**
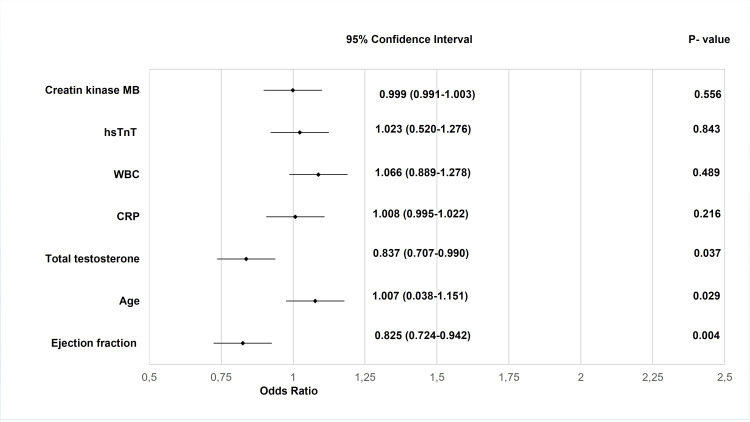
Markers of acute kidney injury in myocardial infarction (univariable regression analysis). hsTnT: High-sensitive cardiac troponin T; hsCRP: High-sensitive C-reactive protein.

The occurrence of AKI was positively predicted by reduced EF, advanced age, and low total T levels (Table 2 and Figures 1-2). Reduced systolic function (OR=0.861; 95% CI=0.758-0.978; P=0.022 for EF) and age (OR=1.094; 95% CI=1.000-1.197; P=0.051) contributed (marginally in the case of age) to the incidence of AKI in a multivariate model. Total T was not an independent factor (OR=0.841; 95%=0.669-1.058; P=0.139) for AKI in AMI.

**Table 2:** Indicators of AKI in male patients with myocardial infarction. AKI: Acute kidney injury; DM: Diabetes mellitus; T: Testosterone; CK: Creatinine kinase; CK-MB: MB isoenzyme of creatinine kinase; hsTnT; High-sensitive cardiac troponin T; hsCRP: High-sensitive C-reactive protein; EF: Ejection fraction; GFRbaseline; Glomerular filtration rate at presentation; GFRmin; Peak reduction in glomerular filtration rate; OR: Odds ratio; CI: Confidence interval.

Male patients	No AKI, n = 42	AKI, n = 13	P-value	OR	95% CI	P-value
Age, years	60.7±12.1	71±8.3	0.019	1.077	1.038-1.151	0.029
DM, n (%)	15 (33.3)	7 (70.0)	0.032			
Anemia, n (%)	9 (21.4)	3 (30.0)	NS			
Statin, n (%)	10 (22.7)	12 (22.2)	NS			
Total T, nmol/l	14.4±5.3	10.3±5.0	0.028	0.837	0.707-0.990	0.037
CK, U/l	1,312.3±1,729.9	1,032.6±780.8	0.621	1.000	0.999-1.000	0.617
CK-MB, U/I	145.2±206.1	105.8±76.9	0.557	0.999	0.991-1.003	0.556
hsTnT, ng/l	2.3±3.1	2.6±2.8	0.847	1.023	0.520-1.276	0.843
hsCRP, mg/l	32.9±45.7	53.4±46.4	0.036	1.008	0.995-1.022	0.216
WBC, x10^9^/l	10.1±3.4	11.0±4.1	0.442	1.066	0.889-1.278	0.489
EF, %	54.3±8.4	44.2±3.4	0.001	0.825	0.724-0.942	0.004
SYNTAX score	15.6±10.8	18.3±5.7	0.096	1.026	0.960-1.096	0.455
GFR_baseline_, ml/min/1.73 m^2^	78.4± 18.8	79.8 ±26.5	0.542	1.011	0.976-1.047	0.535
GFR_min_, ml/min/1.73 m^2^	77.6±18.9	45.3±14	0.001	0.892	0.892-0.959	0.002
Angiographic dye, ml	280±129	337.5±154.8	0.871	1.003	0.996-1.010	0.407

The total T levels were significantly inversely correlated with the peak of hsCRP (r= -0.153; P=0.009) and showed a tendency to inverse relation with the SYNTAX score (r= -0.235; P=0.083).

## Discussion

According to previous animal studies, male sex and androgens are known to confer susceptibility to renal ischemic injury [4,5]. Castration reduces IRI in the kidney. By contrast, ovariectomy does not affect kidney injury induced by ischemia in females [4, 5]. T inhibits the post-ischemic activation of nitric oxide synthase, leading to the expression of inflammatory cytokines (such as TNF-α) and increased functional renal injury [4, 5]. Contrary to the findings in previous animal studies, low levels of plasma total T were consistently correlated with the incidence of AKI in the group of male patients with acute MI in the present study. According to previous reports, the plasma levels of T decrease rapidly and reach a minimum level on the first day of onset of symptoms, and inversely correlate with plasma CRP levels, degree of coronary atherosclerosis, BMI, and prevalence of diabetes mellitus [10-15]. Suppression of androgen production in Leydig cells mediated by inflammatory cytokines is assumed to be a potential mechanism leading to the rapid decrease in T levels during the acute phase of MI [12, 13]. The negative correlation of T with both CRP and the values of the SYNTAX score in the current study indicated a significant association of T with coronary disease severity, presence of acute coronary thrombosis, and, thus, deteriorated left ventricular systolic function. In addition, endogenous T has been previously measured and reported to be abnormally low in male patients with congestive heart failure [16]. Acute decompensated heart failure commonly leads to acute kidney dysfunction (cardiorenal syndrome type 1). Extensive anterior MI, Killip class ≥ 3 and higher central venous pressure are among the most vital indicators for identifying patients with acute MI at high risk of developing AKI [2, 17]. In hospitalized patients, hemodynamic changes leading to reduced cardiac output and renal venous congestion contribute to hypoperfusion of the kidneys and a marked decline in renal function [18]. Inflammation is a key feature of the pathophysiology of IRI [19]. Ischemic renal injury triggers downstream effects such as postischemic leukocyte recruitment, leukocyte-mediated cytotoxicity, and tubule cell injury [19].

In the presence of tissue injury, CRP is rapidly upregulated, being synthesized and secreted from the liver and macrophages mainly in response to circulating IL-6 [20]. The impact of a transient increase in CRP at the time of acute MI is unknown [21]. However, there is convincing evidence that an increase in CRP levels among patients with acute MI may not be merely an epiphenomenon but may contribute directly to the inflammatory injury. High levels of circulating plasma CRP could further augment local macrophage activation, thus exacerbating AKI [22]. In addition, a previous study gave evidence of the long-term prognostic value of increased concentrations of the plasma cytokines IL-8, IL-18, and TNF receptor type 1 for predicting a slower kidney recovery among critically ill patients with AKI [23].

Our results suggested a possible association of AKI with high CRP levels and low plasma total T levels in the acute phase of MI. The present study also observed a negative correlation between CRP and T levels. However, a possible cause-effect association could not be demonstrated upon statistical analysis, probably due to the small number of participants in the study. Heart failure with reduced EF and the severity of coronary disease could strongly reduce the significance of circulating kidney-specific markers of acute injury in contrast to functional (GFR) and non-specific markers (hsCRP and N-terminal pro-B-type natriuretic peptide, [NT-proBNP]) [24]. Accordingly, in our study, low levels of plasma total T were not a significant marker of AKI in a multivariable model.

Delayed CRP clearance could be an unexplored confounding factor in the presented analysis. CRP is primarily metabolized by the liver [25]. Venous renal and liver congestion in cases of low EF and elevated left ventricular filling pressures could also lead to impaired liver clearance [26] and raised plasma levels of CRP. In an observational study, inflammatory biomarkers, including CRP, were elevated in participants with GFR<60 ml/min/1.73 m^2^ vs. GFR≥60 ml/min/1.73 m^2 ^[27]. 

Limitations

Our study has several limitations. First, the number of patients is small, so the results cannot be used for prognostication in greater cohorts. Second, a comparison with a female cohort of patients with acute coronary syndrome could have given a comprehensive notion of the association of the acute testosterone levels with markers of inflammation and AKI. Third, data indicative of subclinical hypogonadism in the past history of our patients have not been collected and recorded. These data would have been helpful in the evaluation of the severity of acute abnormalities in testosterone secretion and their relation to acute MI and AKI. Finally, the dynamics of testosterone levels were not confirmed by repeated measures and thus cannot be evaluated in relation to the renal function in this study.

## Conclusions

In conclusion, the total T levels are significantly inversely related to the peak of hsCRP and a tendency to the SYNTAX score in male patients with acute MI. A low level of plasma total T is not an independent marker of AKI in acute MI. Advanced age and low EF are independent factors for AKI discrimination in a small cohort of patients with acute MI.
